# The Value of Concentrated Foods

**Published:** 1899-10

**Authors:** 


### The Value of Concentrated Foods.

In the second Baillie lecture, delivered at St. George's Hospital by Dr. W. Howship Dickinson on the 18th ult., the subject of "Concentrated Foods" was dealt with by the lecturer in the following terms: "I might make a short chapter like that 'Concerning Snakes in Iceland,' and say that there are no such things. For 'human nature's daily food' are required certain weights of nitrogen, carbon, oxygen and hydrogen which are ultimate forms of matter and not capable of concentration or further reduction to essential principles. An ox can not be got into a teacup by any other process than by leaving the greater part of the animal outside. The stomach of the average man demands every twenty-four hours twenty-one grammes of nitrogen, or about three-quarters of an ounce; 307 grammes of carbon, or about eleven ounces; twelve grammes of hydrogen, not quite half an ounce, besides sulphur and salts. This is required to make up for the waste—to restore what leaves his body in various shapes. Three-quarters of a pound of carbon can not be got into less than three-quarters of a pound. The nutritive effect can not be separated from the bulk. The man must have his pound of flesh. He has to find fuel and to replace waste—charcoal to burn and nitrogen for repairs. If he does not get the necessary poundage he will dwindle, peak and pine. His account of profit and loss must be adjusted according to weight. A drachm of Liebig's extract can not supply more than a drachm. Quality can not supply the place of quantity. The chief nourishers of life's feast are not creatin and creatinin, but albumin, myosin, fat, gluten, starch and sugar. None of these are capable of being administered otherwise than in their natural bodies and corporeal bulk. A sick person who is presented with Valentine's food in teaspoonfuls when he should have milk in pints has as much right to complain as I should have if I were to ask for a steak in the luncheon room and be put off with a minute quantity of Liebig out of the shop. I might object that whatever the virtues of the Liebig, it was not filling at the price. But we can not reckon concentrated extracts as entirely useless, though they are not, properly speaking, food. They contain little or no albumin—some little, some none—no fat, and of the vegetable carbohydrates none. They contain salts and extractives which may be accessories to food, but are not food itself. Perhaps they may be regarded as complementary or supplementary to the essentials of food, and may in special circumstances have special use. I have long been in the habit of using them to help to make up for the loss by suppuration, and so prevent or retard the consequent lardacity. So far as pus carries off the salts, meat extracts can not fail to supply the means of replacing them. These extras are, of course, only to be employed as superadditions to a diet abundant in other respects, not as substitutes for any part of it. They can not take the place of nutriment in bulk. Beside their power of supplying salts and extractives which under special circumstances may be wanting, it is supposed that they have some stimulant action. But the utility of concentrated foods has been greatly exaggerated in practice; they are additions to the essential diet, not substitutes for it. It is possible that a pennyworth of milk may be worth more to the patient than a shilling's worth of Liebig's extract."—*Lancet.*

